# The Role of Apelin/Apelin Receptor in Energy Metabolism and Water Homeostasis: A Comprehensive Narrative Review

**DOI:** 10.3389/fphys.2021.632886

**Published:** 2021-02-10

**Authors:** Gonghui Hu, Zhen Wang, Rumin Zhang, Wenping Sun, Xiaoyu Chen

**Affiliations:** ^1^Department of Physiology, Shandong First Medical University (Shandong Academy of Medical Sciences), Taian, China; ^2^Neurobiology Institute, Jining Medical University, Jining, China; ^3^Department of Pathology, Shandong First Medical University (Shandong Academy of Medical Sciences), Taian, China

**Keywords:** apelin, apelin receptor, signal transduction, energy metabolism, water homeostasis

## Abstract

The apelin receptor (APJ) is a member of the family A of G-protein-coupled receptors (GPCRs) and is involved in range of physiological and pathological functions, including fluid homeostasis, anxiety, and depression, as well as cardiovascular and metabolic disorders. APJ was classically described as a monomeric transmembrane receptor that forms a ternary complex together with its ligand and associated G proteins. More recently, increasing evidence indicates that APJ may interact with other GPCRs to form heterodimers, which may selectively modulate distinct intracellular signal transduction pathways. Besides, the apelin/APJ system plays important roles in the physiology and pathophysiology of several organs, including regulation of blood pressure, cardiac contractility, angiogenesis, metabolic balance, and cell proliferation, apoptosis, or inflammation. Additionally, the apelin/APJ system is widely expressed in the central nervous system, especially in neurons and oligodendrocytes. This article reviews the role of apelin/APJ in energy metabolism and water homeostasis. Compared with the traditional diuretics, apelin exerts a positive inotropic effect on the heart, while increases water excretion. Therefore, drugs targeting apelin/APJ system undoubtedly provide more therapeutic options for patients with congestive heart failure accompanied with hyponatremia. To provide more precise guidance for the development of clinical drugs, further in-depth studies are warranted on the metabolism and signaling pathways associated with apelin/APJ system.

## Introduction

The apelin receptor (APJ) was first identified as an orphan G protein-coupled receptor (GPCR) in 1993, with the closest identity to the angiotensin II (Ang II) receptor, type AT1a (O’Dowd et al., 1993). APJ belongs to family A of the GPCRs (O’Dowd et al., 1993), and is a potential pharmacotherapeutic target for heart failure, hypertension, and other cardiovascular diseases. APJ shares 31% identity with the amino acid sequence of the human AT1 receptor, and its hydrophobic transmembrane region shares 54% identity with human AT1 (O’Dowd et al., 1993), while it does not bind to members of the angiotensin family (De Mota et al., 2000). APJ remained an orphan receptor until 1998 when Tatemoto et al. (Tatemoto et al., 1998; Lee et al., 2000) identified a 36-amino acid peptide termed Apelin, for APJ endogenous ligand.

## Biological Characteristics of Apelin/APJ System

### Tissue Distribution and Receptor Binding

**Figure 1:**
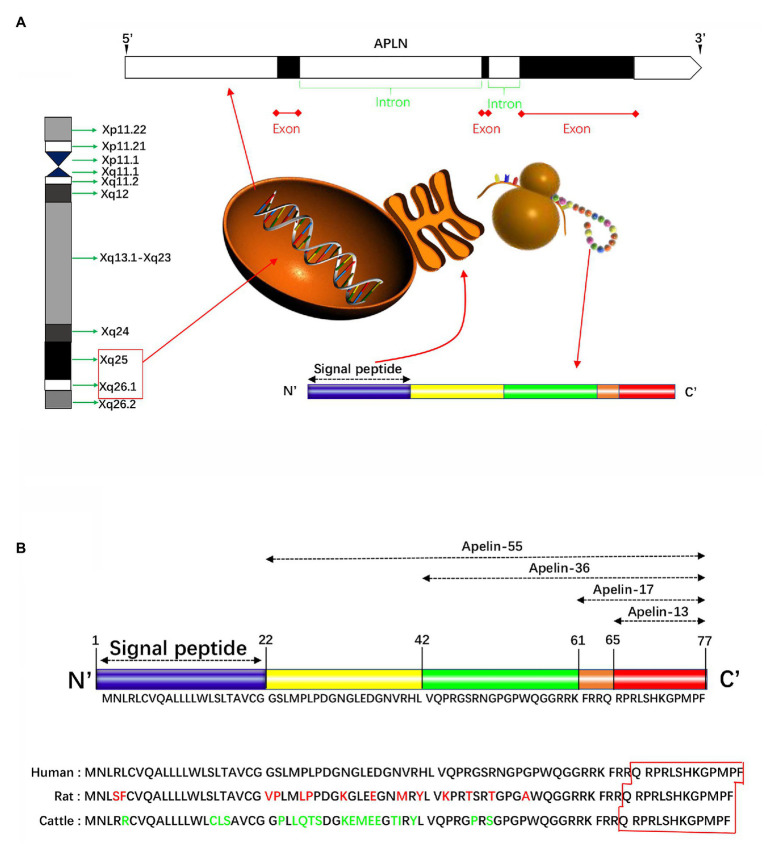
Schematically presentation of apelin in gene and protein. **(A)** The human apelin gene is located in the chromosome Xq25-26.1. Its gene sequence contains three exons and two introns. The apelin gene encodes a 77-amino acid propeptide, preapelin, which has a hydrophobic N-terminal region and a C-terminal. The N-terminal is a signal sequence, and the C-terminal has a variety of biological activities and specific regions that bind to apelin receptor (APJ). **(B)** The N-terminal residue of preapelin is modified by endopeptidase to form pro-apelin-55, which is subsequently cleaved into various forms, including apelin-13, apelin-17, and apelin-36. The results of the cohort study of the amino acid sequences of cattle, humans, and rats showed that the 17 amino acids at the C-terminal were extremely conserved. Apelin-13 is the shortest active fragment present in organisms. Apelin-36 has 86–100% structural homology between human, cattle, and rat, while the C-terminal sequence homology up to 100%.

The human apelin gene, namely APLN, is localized on the chromosome Xq25-26.1 (Figure 1A). Its gene sequence contains three exons and two introns (Lee et al., 2000), the coding region spans two exons 1 and 2, and the 3' untranslated region also spans two exons 2 and 3, thereby explaining the existence of two transcription products with different sizes in different tissues (O’Carroll et al., 2000). Apelin gene in rats and mice, known as Apln, is located at chromosomal locations Xq35 and XA3.2, respectively. Apelin encodes a 77-amino acid prepropeptide, and amino acid sequence of apelin is similar to that of Ang-II. Bovine, human, rat, and mouse preproapelin precursors have 76–95% homology and appear to exist endogenously as a dimeric protein (Medhurst et al., 2003). The results of aligned studies based on amino acid sequences of cattle, humans, rats, and mice showed that 17 amino acids of apelin at the C-terminal (Lys-Phe-Arg-Arg-Gln-Arg-Pro-Arg-Leu-Ser-His-Lys-Gly-Pro-Met-Pro-Phe, apelin-17) were extremely conserved (Galanth et al., 2012). Other experimental results showed that tryptophan at position 55 and phenylalanine at position 77 were highly conserved among different species (Medhurst et al., 2003). The N-terminal residue of preapelin is post-translationally modified by endopeptidase to form pro-apelin-55, which is subsequently cleaved into various forms (Tatemoto et al., 1998), including apelin-13 (65–77), apelin-17 (61–77), and apelin-36 (42–77; Figure 1B). Recently, *in vitro* studies have shown that the pro-protein convertase subtilisin/kexin type 9 (PCSK9) can cleave pro-apelin-55 directly into apelin-13 without producing longer isoform peptides (Flahault et al., 2017). The biological activity of apelin is inversely proportional to its length, in which the efficacy of apelin-17 is higher than that of apelin-36, while lower than that of apelin-13 (Bełtowski, 2006). Translated N-terminal glutamine residues of apelin-13 are catalyzed by glutamine cyclase to modify pyroglutamidation to produce the pyroglutamide form of apelin-13 [pyr^1^]-apelin-13, which can prevent it from degradation by exopeptidase, thereby exerting more long-term biological activities (Habata et al., 1999). Besides, [pyr^1^]-apelin-13 has been broadly used to study *in vivo* and *in vitro* reactions, and is considered as a physiological ligand for APJ due to its higher anti-degradation properties (Habata et al., 1999; Maguire et al., 2009; Azizi et al., 2013).

Apelin is abundantly distributed in the central nervous system (CNS) and peripheral tissues in humans and rodents. In the human’s CNS, apelin is found in the dorsal raphe nucleus, amygdala, and hypothalamus (De Mota et al., 2004). Apelin is also highly expressed in the thalamus and frontal cortex of CNS, and is relatively less expressed in the hypothalamus, midbrain, caudate nucleus, and substantia nigra-amygdala connections. The expression of apelin was also detected in the spinal cord and pituitary (De Mota et al., 2000; Lee et al., 2000; O’Carroll et al., 2000). Moderate expression of apelin was previously detected in the heart, liver, kidney, gastrointestinal (GI) tract, adipose tissue, ovary, and testis in peripheral tissues (Edinger et al., 1998; Hosoya et al., 2000; Lee et al., 2000; Kawamata et al., 2001; Medhurst et al., 2003; Kleinz and Davenport, 2005), and is highly expressed in lung and breast (Kawamata et al., 2001). In rats, preproapelin and APJ mRNA were detected in peripheral tissues, including testis, intestine, kidney, and in the fetus. In peripheral tissues, apelin is expressed at the highest level in the placenta, highly in the heart, lung, kidney, and vascular endothelial cells, while its expression is relatively low in adipose tissues, connective tissues, and vascular smooth muscle cells (VSMCs; O’Carroll et al., 2000).

The human APJ gene, known as APLNR, is localized on autosomal 11q12 and encodes 380 amino acid proteins (O’Dowd et al., 1993). APJ gene in rats and mice, known as Aplnr, is located at chromosomal locations 3q24 and 2E1, respectively, and both encode 377 amino acid proteins (O’Carroll et al., 2013). The ApelinR amino acid sequence is conserved across species, with more than 90% of homology between human and rodents, and up to 50% of homology with other non-mammalian species, such as zebrafish or frog (O’Dowd et al., 1993; Devic et al., 1996). The gene encoding APJ is intronless (O’Dowd et al., 1993). Glu20 and Asp23 at the extracellular N-terminus of APJ are the first physiological sites found to bind to the ligand apelin and may play substantial roles (Zhou et al., 2003a,b). Thereafter, direct mutation of binding sites by a three-dimensional molecular model demonstrated that Asp94, Glu174, and Asp284 could also be involved in the binding of human apelin (Asp.$1092, Glu 172, and Asp.$10282 in the rat sequence; Gerbier et al., 2015). APJ is not a subtype of the angiotensin receptor and cannot bind to angiotensin to exert biological effects. Besides, β-arrestin-related phosphorylation, palmitoylation, and glycosylation sites at the C-terminal of APJ have been experimentally confirmed (O’Dowd et al., 1993), and these sites are essential for the phosphorylation and endocytosis of APJ.

*In situ* hybridization and immunohistochemistry showed that APJ is extensively expressed in the CNS, including supraoptic nucleus, paraventricular nucleus, midbrain, basal ganglia, hippocampus, and spinal cord (O’Carroll et al., 2013). In particular, APJ and arginine vasopressin (AVP) are co-expressed in neuronal cells in the supraoptic and paraventricular nuclei of the hypothalamus (De Mota et al., 2004; Reaux-Le Goazigo et al., 2004; Bodineau et al., 2011). Furthermore, a moderate APJ mRNA expression was found in the renal collecting duct (CD; Hus-Citharel et al., 2008) where the AVP V2 receptor (V2R) are also expressed (Ostrowski et al., 1992). This strongly strengthens that apelin may regulate body fluid balance with AVP (Roberts et al., 2010). The distribution of APJ in the peripheral tissues is similar to that of apelin, with the highest expression in the placenta and spleen, and a relatively low expression in the heart, lung, liver, kidney, and GI tract (O’Carroll et al., 2000). APJ can also be expressed in interleukin-2 (IL-2)-activated peripheral blood mononuclear cells (Bełtowski, 2006).

### Apelin/APJ System-Associated Signaling Pathways

**Figure 2:**
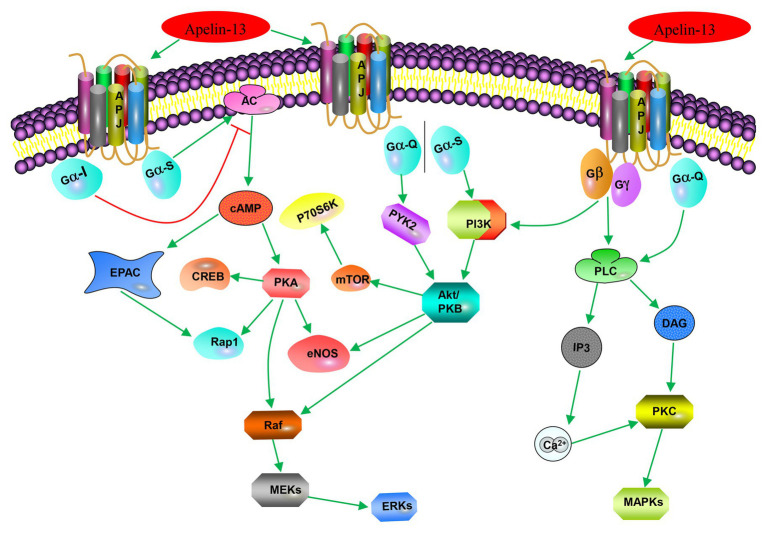
Apelin-associated signaling pathways. Apelin-13 (in addition to apelin-17, apelin-36, and [pyr^1^]-apelin-13) binds to APJ to couple Gαi, inhibits forskolin (FSK)-induced cAMP generation, and blocks various biological effects produced by the protein kinase A (PKA) pathway; it (or apelin-36) binds to APJ-coupled Gio or Gq/11 to activate extracellular signal-regulated kinase 1/2 (ERK1/2) through protein kinase C (PKC) pathway; it (or [pyr^1^]-apelin-13) activates phosphorylation of ribosomal protein S6 kinase (p70S6) through the PI3K/Akt and ERK1/2 signaling pathways. Apelin-13 promotes cell proliferation, division, migration, and metabolic function through the PI3K/Akt signaling pathway or MAPK pathway; and it (or apelin-12) stimulates endothelial nitric oxide synthase (eNOS) through the protein kinase B (PKB) pathway.

The apelin/APJ system mediates signal transduction mainly by coupling to G protein (Figure 2). Apelin-36, apelin-13, apelin-17, and [pyr^1^]-apelin-13 block various biological effects generated by protein kinase A (PKA) pathway (Habata et al., 1999; De Mota et al., 2000; Medhurst et al., 2003; El Messari et al., 2004) by inhibiting forskolin (FSK)-induced cyclic adenosine monophosphate (cAMP) production, suggesting that Gαi/o protein is involved in apelin/APJ system-associated signaling pathway. The APJ-coupled Gαi protein was first confirmed by determining the extracellular acidification rates of apelin-13 and apelin-36 (Tatemoto et al., 1998). Further experiments on phosphorylated inhibition of adenylate cyclase and extracellular signal-regulated kinase 1/2 (ERK1/2) demonstrated that murine apelin receptor is preferentially coupled to Gαi1 and Gαi2, rather than Gαi3 in Chinese hamster ovary cells (Masri et al., 2006). Apelin activation of ERK1/2 is mediated by protein kinase C (PKC), indicative of coupling to either Gαi or Gαq/11 (Masri et al., 2006). However, the positive inotropic effect induced by apelin in rats is only partially counteracted by pertussis toxin (PTX) and PKC inhibitors. The results indicate that some effects of APJ may be realized by double-coupling PTX-sensitive Gi/o protein and PTX-insensitive Gq/11 protein (Szokodi et al., 2002). The same results were also confirmed in APJ of adipocytes (Szokodi et al., 2002). In human umbilical vein endothelial cells (HUVECs), activated APJ binds to Gαi3 in an apelin-independent manner, resulting in phosphorylation of two histone deacetylases (HDAC) 4 and HDAC5, producing cytoplasmic translocation, as well as activating the transcription factor myocyte enhancer factor 2 (MEF2; Kang et al., 2013). The activation of apelin/APJ system can also trigger a cascade of intracellular signaling molecules, including phosphatidylinositol 3-kinase/protein kinase B (PI3K/Akt), P70 ribosomal protein S6 kinase (P70S6K), nitric oxide synthase (NOS), AMP-activated protein kinase (AMPK), reactive oxygen species (ROS), and other signaling pathways, ultimately targeting transcription factors to regulate cellular function (Kleinz and Baxter, 2008; D’Aniello et al., 2009).

**Figure 3:**
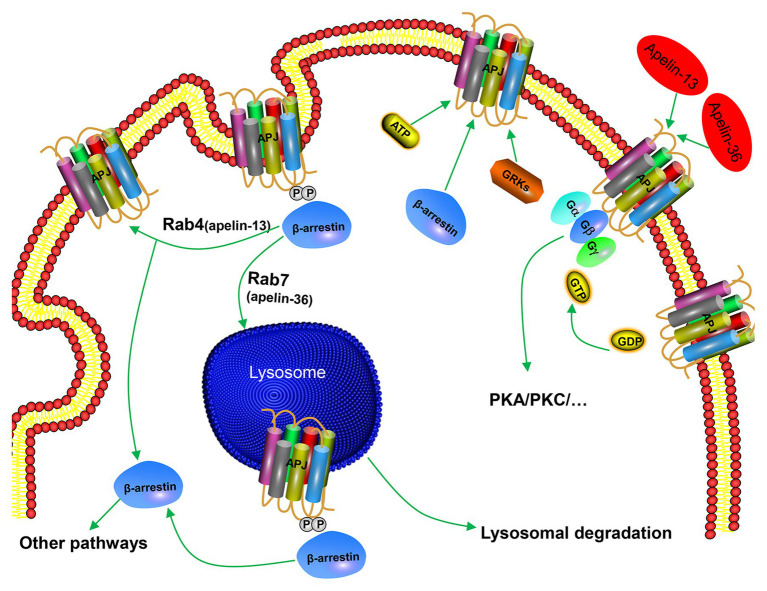
Mechanism of β-arrestin-dependent APJ desensitization and endocytosis. Apelin-36 binds to APJ and forms APJ/apelin-36 complex, which is localized to intracellular lysosome through Rab7 signaling pathway, leading to persistent desensitization of APJ, while apelin-13 forms APJ/apelin-13 complex with APJ and then rapidly circulates APJ to the cell surface through Rab4 signaling pathway, leading to transient desensitization of APJ.

In addition to G-protein-dependent intracellular signal transduction pathways, apelin activates APJ and mediates a receptor’s desensitization and endocytosis through β-arrestin-dependent signaling pathway. Apelin-36 and apelin-13 induce endocytosis and desensitization of APJ in two ways. Apelin-36 activates APJ to recruit β-arrestin, and β-arrestin enters the endocytosis process together with the receptor, and localizes to the intracellular lysosome through Rab7 signaling pathway, resulting in persistent desensitization of APJ (Figure 3). However, apelin-13 activates APJ to recruit β-arrestin instantaneously. Afterward, endocytosis of the APJ by apelin-13 is characterized by dissociation from β-arrestin and rapid recycling to the cell surface through Rab4 signaling pathway, leading to APJ transient desensitization (Figure 3). Both endocytosis and desensitization require the involvement of β-arrestin (Masri et al., 2006; Lee et al., 2010). This indicates that pharmacological properties of apelin isoforms are slightly different, which may produce different physiological effects. It has been found that apelin-K16P activates intracellular G-protein-dependent signaling pathways, while it does not recruit β-arrestin to bind to APJ and endocytosis. Therefore, apelin-K16P exhibits a biased ligand property. Further study revealed that apelin-K16P lost its ability to reduce blood pressure in rats (El Messari et al., 2004), thus, it can be confirmed that reduction of blood pressure by inducing apelin may be associated with β-arrestin-dependent signaling pathway. Our experimental findings indicated that mutation in the ser^348^ of APJ did not influence the activation of G protein and downstream signaling pathways of G protein, although resulted in inactive β-arrestin (Chen et al., 2014). Therefore, the APJ-S348A mutant exhibits the characteristics of a biased receptor.

As a member of the GPCR superfamily, APJ can also form heterodimer structures with other GPCRs and participate in different biological effects, in addition to acting as a monomer. Immunoprecipitation and fluorescence resonance energy transfer (FRET) experiments showed that the dimerization of APJ and Ang-II receptor inhibited the Ang-II-associated signaling pathways, and played a role in the Ang-II-mediated animal models of atherosclerosis (Chun et al., 2008). Previous studies conducted in our laboratory confirmed a heterodimer between APJ and κ-opioid receptor, which increased ERK phosphorylation by activating PKC and inhibited the activity of PKA, thereby promoting cell proliferation (Li et al., 2012). Besides, APJ can form a heterodimer with bradykinin type 1 receptors in HUVECs, the heterodimer can upregulate PKC activity and intracellular Ca^2+^ concentration by coupling Gαq, resulting in increased phosphorylation of endothelial nitric oxide synthase (eNOS) and cell proliferation (O’Carroll et al., 2013). However, the biased effects of GPCR dimer on signaling pathway and changes in biological properties have still remained elusive.

## Apelin and Glucose Metabolism

Nowadays, evidence has been increasing that apelin is closely related with glucose metabolism and insulin sensitivity. In this review, we summarize the role of apelin/APJ system in peripheral tissues and CNS. Nevertheless, the effects of apelin on glucose homeostasis in models with diet-induced obesity, at present, do not appear clearly coordinated between peripheral tissues and CNS. In conclusion, apelin/APJ system becomes a potential therapeutic target in promoting glucose uptake, increasing overall glucose utilization and ameliorating insulin resistance, although hazardous effects may arise on the body due to aberrant apelin concentrations.

### Regulation of Glucose Metabolism by Apelin in Periphery Tissues

The apelin/APJ system plays a pivotal role in glucose metabolism and insulin sensitivity. Low-dose intravenous (IV) apelin can reduce blood glucose and improve glucose tolerance in normal mice. When hepatic glucose production is fully inhibited during hyperinsulinemic-euglycemic clamp, apelin can reduce blood glucose by increasing glucose uptake of skeletal muscles and adipose tissues, thereby promoting overall glucose utilization (Castan-Laurell et al., 2011; Bertrand et al., 2015; He et al., 2019).

Skeletal muscle plays a fundamentally important role in the maintenance of normal glucose homeostasis and in regulating whole-body carbohydrate metabolism. Apelin can promote glucose transport (Dray et al., 2008; Bertrand et al., 2015) in isolated soleus muscle and synergize with insulin. Studies have found that apelin’s effect on reducing glucose level depends on the activation of AMPK and eNOS (Dray et al., 2008; Bertrand et al., 2015). However, it has been reported that apelin promotes glucose uptake mainly through AMPK pathway rather than eNOS pathway in cultured C2C12 muscle cells (Yue et al., 2010). Besides, apelin can increase Akt phosphorylation in isolated C2C12 myotubes (Xu et al., 2012; Bertrand et al., 2015). It is noteworthy that plasma apelin concentration was increased in obese and insulin-resistant mice fed with high-fat diet, IV injection of apelin during hyperinsulinemic-euglycemic clamp had still remained effective in improving insulin sensitivity (Castan-Laurell et al., 2011). Therefore, exogenous administration of apelin can significantly improve glucose tolerance and insulin sensitivity, mainly depending on the improvement of metabolic functions of skeletal muscles, in addition to elevation of skeletal muscle glucose uptake. It was found that apelin knockout mice exhibited an enhanced insulin resistance during high-fat diet feeding, which further confirmed the role of apelin in the regulation of glucose homeostasis (Yue et al., 2010, 2011). In human adipose tissue explants, apelin can stimulate glucose transport in an AMPK-dependent manner, whereas 3 T3-L1 adipocytes can achieve the same outcome by activation of PI3K/Akt signaling pathway. In addition, apelin also increased insulin-stimulated glucose transport in 3 T3-L1 cells with insulin-resistant (Zhu et al., 2011).

In addition to skeletal muscles and adipose tissues, apelin increased glucose uptake and Glut4 membrane translocation in the myocardium of C57BL/6 J mice *in vivo* (Xu et al., 2012; Bertrand et al., 2015). According to the results of *in vitro* experiments, apelin also increased glucose transport in embryonic cardiomyocyte cell line H9C2 (Xu et al., 2012; Bertrand et al., 2015).

Glucose can rapidly stimulate the secretion of apelin in mouse intestinal epithelial cells. Apelin increases the net glucose flow of GI mucosal barrier and enhances the glucose transport from intestinal lumen to blood by promoting AMPK phosphorylation, regulating the ratio of sodium-glucose co-transporter 1 (SGLT-1) to glucose transporter 2 (GLUT2). Conversely, administration of the apelin blocker can reduce hyperglycemia after oral glucose administration. Glucose stimulates the secretion of apelin in intestinal epithelial cells, and apelin enhances the glucose transport from intestinal lumen to blood. The interreaction between glucose and apelin leads to the increase of portal vein glucose levels, which in turn stimulates the rapid secretion of insulin and improves insulin sensitivity (Fukaya et al., 2007; Delaere et al., 2010; Dray et al., 2013). Therefore, apelin can also maintain the homeostasis of glucose by promoting glucose absorption of intestinal epithelium, thereby increasing portal blood glucose and insulin secretion.

In the GI tract, the enteric nervous system (ENS) is involved in various physiological functions, including modulation of intestinal contraction. The ENS is essentially composed of excitatory motor neurons [choline acetyl transferase (ChAT)] or inhibitory motor neurons [neuronal NOS (nNOS)] that stimulate or inhibit intestinal contractility and motility. During diabetes, there is a loss of inhibitory neurons, in addition to an increase in cholinergic innervations in the proximal part of the intestine (Chandrasekharan and Srinivasan, 2007). The gut-brain axis is considered as a major regulatory checkpoint in the control of glucose homeostasis. The detection of nutrients and/or hormones in the duodenum informs the hypothalamus of the host’s nutritional state (Duparc et al., 2011b; Breen et al., 2013). This process may occur *via* hypothalamic neurons, modulating release of nitric oxide (NO), which in turn controls glucose entry into tissues (Duparc et al., 2011a; Knauf et al., 2013). The effects of apelin on glucose metabolism in the gut-brain axis were experimentally investigated by Fournel et al. (2017) in normal and obese/diabetic mice, glucose utilization is improved by the decrease of ENS-evoked duodenal contraction activities in response to apelin, causing an increase in hypothalamic release of NO. Consequently, skeletal muscle glucose uptake increases substantially during dynamic exercise. A novel mode of communication between the intestine and the hypothalamus that controls glucose utilization has been identified, in addition to presentation of oral apelin administration as a novel potential target to treat metabolic disorders.

### Regulation of Glucose Metabolism by Apelin in CNS

Apelin mRNA widely exists in different nuclei of the CNS and participates in the regulation of glucose metabolism (Castan-Laurell et al., 2011). It is well-known that hypothalamus is a key site of action for leptin-mediated control of glucose metabolism. In response to different stimuli, such as nutrients, hormones, neuropeptides, and stress, it can affect glucose homeostasis by a variety of pathways (Yamada and Katagiri, 2007). Apelin-positive nerve fibers in the hypothalamus indicate the existence of apelin-synthesizing neurons, confirming the role of apelin in circulating peptide, as well as being a neurotransmitter.

Experiments have shown that blood glucose level of mice fed with normal diet was dramatically decreased after intraventricular injection of low-dose apelin-13 for within 60–210 min, whereas significantly improved glucose tolerance and insulin tolerance. The release of NO and the active phosphorylated form of eNOS rapidly increased within 2–15 min in the hypothalamic sections of mice after treatment with low-dose apelin-13. After further knocking out eNOS in mice, it was found that the regulatory effect of central apelin on glucose homeostasis disappeared, including blood glucose and glucose tolerance. Therefore, apelin may regulate glucose homeostasis and promote glucose uptake and utilization through NO-dependent pathways in mice fed with normal diet (Duparc et al., 2011a). In obese or diabetic mice fed with high-fat diet, the regulatory effect of central apelin on glucose homeostasis was attenuated (Clarke et al., 2009; Duparc et al., 2011a). After fasting, low-dose apelin-13 was injected into the lateral ventricle of obese diabetic mice fed with high-fat diet, and no changes were measured in blood glucose level, insulin level, glucose tolerance, and insulin tolerance (Duparc et al., 2011a). This may be due to the stimulation of hypothalamus by peripheral apelin (Knauf et al., 2013). At present, it is elusive whether peripheral apelin can reach hypothalamus and regulate central apelin level. However, it was previously found that the concentration of apelin in the hypothalamus increased after its intraperitoneal (IP) injection (Higuchi et al., 2007; Castan-Laurell et al., 2011).

After high-dose injection of apelin-13 into the lateral ventricle of mice fed with normal diet, it was noted that both peripheral blood glucose and insulin level were elevated. The hyperinsulinemic-euglycemic clamp experiment showed that intracerebroventricular (ICV) injection of high-dose apelin could reduce systemic sensitivity to insulin and produce insulin resistance. However, high-dose apelin-13 was injected into the lateral ventricle in the obese diabetic mice fed with high-fat diet, which showed that blood glucose level was significantly increased, while a slight change in insulin level was observed. Meanwhile, in the hypothalamic explant experiment, NO release content and eNOS phosphorylation form of obese diabetic mice did not change in either normal feeding or fasting state, in presence of low- or high-dose ICV injection (Duparc et al., 2011a). These results suggest that central apelin-dependent NO signaling pathway is impaired in pathological conditions, e.g., obesity or diabetes, or there are other pathways regulating glucose metabolism in the CNS. A number of scholars (Drougard et al., 2014) demonstrated that ICV injection of apelin can activate the sympathetic nervous system through ROS-related signaling pathway to promote hepatic glycogen breakdown and gluconeogenesis, thereby leading to the increase of fasting blood glucose level. This highlighted the regulatory effect of central apelin on glucose metabolism under pathological conditions, and also indicated that the liver plays a major role in the control of glucose homeostasis (Knauf et al., 2013; Chaves-Almagro et al., 2015).

There are several other regulatory pathways of apelin on central glucose metabolism, such as apelin binding to APJ, and the resultant separation of Gαq heterotrimers triggers Gβγ-dependent activation of phospholipase C-β (PLC-β) signals that can inhibit M-currents, thereby leading to depolarization of pro-opiomelanocortin (POMC) neurons (Lee et al., 2015). POMC neurons express apelin receptor mRNA, and K17F strongly increases release of α-Melanocyte-stimulating hormone (α-MSH) from rat hypothalamic explants, whereas the inactive apelin fragment R10F dose not. This suggests that apelin may be somatodendritically or axonally released from POMC neurons, stimulating the release of α-MSH in an autocrine manner, which is a neuropeptide reducing food intake, as well as inducing weight loss (Fan et al., 1997; Grill et al., 1998). Goazigo et al. also reported an increase in hypothalamic apelin level in obese db/db mice, obese fa/fa Zucker rats, and in high-fat diet-fed mice (Reaux-Le Goazigo et al., 2011; Knauf et al., 2013). Therefore, the apelin from POMC neurons may be involved in regulating the pathological conditions of diabetic patients. The activity of POMC neurons may also be enhanced by the production of ROS (Qiu et al., 2010; Diano et al., 2011; Qiu et al., 2014). Therefore, apelin is widely involved in the glucose metabolism at the central level, and it dose-dependently possesses a variety of beneficial or detrimental effects (Fan et al., 1997; Grill et al., 1998; Clarke et al., 2009; Duparc et al., 2011a).

## The Role of Apelin in Lipid Metabolism and Thermogenesis

To date, a limited number of studies have concentrated on the role of apelin in lipid metabolism. In isolated adipocytes and differentiated 3 T3-L1 adipocytes, apelin can inhibit lipolysis *via* a pathway involving Gq, Gi, and AMPK in isolated rodent cells and mature 3 T3-L1 adipocytes (Castan-Laurell et al., 2011; Bertrand et al., 2015). Apelin can be activated by AMPK and it reduces the release of free fatty acid (FFA) from 3 T3-L1 adipocytes by increasing the amount of peripheral lipoproteins surrounding lipoproteins, making them more stable and resistant to lipase. However, apelin has no influence on basal or isoproterenol-stimulated lipolysis in human adipose tissue explants or isolated adipocytes. Treatment of standard and high-fat diet-fed mice with apelin had shown influences on adipose tissue lipolysis (Attané et al., 2011; Yue et al., 2011; Than et al., 2012). Daily IP injection of apelin for 2 weeks has been found to reduce triglyceride content and fat deposition in adipose tissue at different sites in standard and high-fat diet-fed mice, including a significant reduction in liver triglyceride content and expressions of different adipogenesis-related genes. The treatment procedure did not affect food intake, while it increased rectal temperature and oxygen consumption (Yamamoto et al., 2011).

After treatment with cyclooxygenase-2 (COX-2) inhibitor, the vascular function of apelin gene knockout mice fed with high-fat diet was improved, and the content of fat mass was reduced (Chaves-Almagro et al., 2015). The results demonstrated that the weight gain of apelin knockout mice may be related to the increased vascular permeability of adipose tissue, leading to more fatty acid absorption. Therefore, apelin may also prevent the development of obesity by maintaining vascular integrity (Hwangbo et al., 2017). Recent studies have shown that inactivation of the forkhead transcription factor 1 and inhibition of endothelial expression of fatty acid binding protein 4 (FABP4) are key downstream signaling targets of apelin/APJ system. Both apelin- and APJ-knockout mice showed an increased expression of endothelial FABP4 and accumulation of excess fatty acids. Treatment of APJ knockout mice with FABP4 inhibitor inhibited the accumulation of fatty acids, and effectively prevented lipid metabolism disorders (Hwangbo et al., 2017). These findings broaden the prospects for the treatment of type 2 diabetes and lipid metabolism disorders (Chaves-Almagro et al., 2015; Hwangbo et al., 2017).

Experiments focused on heart of mice fed with high-fat diet with aortic ligation exhibited an attenuated cardiac systolic function (Chaves-Almagro et al., 2015). After 28 days of treatment, apelin not only reversed cardiac hypertrophy but also prevented the reduction of fatty acid and glucose oxidation induced by aortic ligation. Further isolation of mouse cardiomyocytes showed (Chaves-Almagro et al., 2015) that apelin increased fatty acid oxidation dependent on sirtuin-3 activation. In addition, the expressions of peroxisome proliferator-activated receptor-γ coactivator 1α (PGC1α), nuclear respiratory factor 1 (NRF-1), and mitochondrial transcription factor A (TFAM) genes were also elevated, which was consistent with apelin’s effects on mitochondrial biogenesis of skeletal muscle cells.

In skeletal muscle cells, chronic apelin treatment, in obese and insulin-resistant mice, was also shown to increase fatty acid oxidation in muscles through activation of AMPK pathway, and it reduced the rate of incomplete oxidation of long-chain acylcarnitine related to insulin (Alfarano et al., 2015). In addition to promotion of lipid utilization, apelin can also increase mitochondrial biogenesis of skeletal muscle cells by increasing the expression of PGC1α (Bertrand et al., 2015). This sustained effect is associated with increased expressions of PGC1α, NRF-1, and TFAM, which can enhance the oxidative phosphorylation of mitochondria and mitochondrial biogenesis (Chaves-Almagro et al., 2015).

Energy expenditure in response to apelin treatment has also been studied *via* thermogenesis (Drougard et al., 2016). Brown adipose tissue (BAT) has a high density of mitochondria with high amounts of mitochondrial uncoupling protein 1 (UCP1), allowing the uncoupling of fatty acid oxidation from ATP production to generate heat (Knauf et al., 2008; Lam et al., 2009). The higher rectal temperature and oxygen consumption in apelin-treated mice may be correlated to the increased expression of UCP1 in BAT (Castan-Laurell et al., 2011; Bertrand et al., 2015). Furthermore, apelin/APJ signaling promotes brown adipocyte differentiation by increasing the expressions of brown adipogenic and thermogenic transcription factors *via* the PI3K/Akt and AMPK signaling pathways. Apelin can alleviate the inhibitory effect of TNF on brown adipogenesis. Apelin can also increase the basal activity of brown adipocytes by increasing the expressions of PGC1 and UCP1, mitochondrial biogenesis, and oxygen consumption (Than et al., 2015). In fact, the role of apelin in the control of thermogenesis remains to be unraveled, and is complex. Chronic ICV injection of apelin decreased the level of PGC1α mRNA in BAT that is a key regulator of mitochondrial biogenesis (Puigserver, 2005), and PR domain containing 16 (PRDM16) mRNA, a key transcriptional cofactor concerned in BAT differentiation (Seale et al., 2007). This result suggests that chronic ICV apelin perfusion decreases mitochondrial biogenesis and reduces BAT activity, which can justify the alteration of thermogenesis in mice. In addition, UCP1 protein level is significantly decreased in BAT in response to chronic ICV apelin perfusion, confirming disruption of its function. This was associated with a decrease in body temperature and an altered response to cold exposure in mice perfused with chronic ICV apelin. These molecular modifications involved in BAT are strongly associated with the decrease in rectal temperature (Drougard et al., 2016), thereby contributing to explain the decreased energy expenditure upon apelin treatment. These data provide compelling evidence that central apelin contributes to the development of type 2 diabetes by altering energy expenditure, thermogenesis, and fat browning. At the same time, both *in vivo* and *in vitro* experiments confirmed that apelin can increase brown-like characteristics in white adipocytes, which provides a potential therapeutic approach to combat obesity and metabolic disorders (Boucher et al., 2005; Than et al., 2015).

## The Relationship Between Apelin and Food Intake

Regarding the role of apelin in the control of food intake, its precise function requires further assessment. Previous studies examined the effects of IV and ICV injections of apelin-13 on food intake in Wistar rats. ICV injection of 1 and 3 nmol of apelin-13 resulted in a reduction in food intake in both fed and fasted rats. However, IV injection of 10 nmol of apelin-13 did not cause any change in food intake in either fed or 24-h fasted rats (Sunter et al., 2003). Lin et al. (2014) performed IP injection of apelin-13 and studied food intake in injected fish. Apelin injected at a dose of 100 ng/g body weight induced a significant increase in food intake compared with saline injected into fish. The results suggest that apelin acts as an orexigenic factor in Ya-fish (Lin et al., 2014). Consistent with previously reported results, Valle et al. (2008) assessed the effects of a chronic 10-day ICV infusion of apelin-13 into the third ventricle on food intake in C57BL/6 mice. Apelin-13 (1 μg/day) significantly increased food intake on days 3–7 of infusion; thereafter, food intake in treated and control individuals converged. Such discrepancies could be justified by the injected quantity of apelin into the brain, the route of injection, and animal species (Chaves-Almagro et al., 2015). Further investigation on the effects of apelin on food intake may provide new ideas for the treatment of obesity.

## The Relationship Between Apelin and Water Metabolism

### Regulatory Effects of Apelin/APJ System on Water Metabolism in CNS

The expression of apelin/APJ in magnocellular neurons of the hypothalamus and periventricular organelles suggests that it may be involved in the regulation of body fluid balance. Apelin-immunoreactive neurons are particularly abundant in hypothalamic nuclei, i.e., the supraoptic nucleus (SON), paraventricular nucleus (PVN), and arcuate nucleus. AVP, also known as an antidiuretic hormone, is involved in the maintenance of body fluid balance by regulating water reabsorption, as well as influencing urine volume (Reaux et al., 2001; Brailoiu et al., 2002). By using double-color immunofluorescence staining, it was revealed that AVP and apelin, as well as their respective receptors, are co-localized in the magnocellular neurons of the hypothalamus (De Mota et al., 2004; Reaux-Le Goazigo et al., 2004; Bodineau et al., 2011), suggesting an interaction between apelin and AVP in regulating the water balance in the body.

In animal models, apelin-17 injected into the third ventricle exerted a diuretic effect owing to inhibition of the discharge activity of AVP neurons and the release of AVP (De Mota et al., 2004; Gerbier et al., 2017). Similarly, in lactating rats with significantly increased AVP synthesis and release, ICV injection of apelin-17 resulted in a notable reduction in AVP release. These findings demonstrate that apelin is a natural inhibitor that can participate in controlling body fluid homeostasis by regulating the discharge activity of AVP neurons and AVP release *in vivo*.

The results of water deprivation showed that activation of rat AVP neurons by dehydration stimulation resulted in the release of AVP faster than its synthesis rate, increased plasma AVP concentration, and depletion of stored AVP in hypothalamic neurons (De Mota et al., 2004; Reaux-Le Goazigo et al., 2004). It is noteworthy that the increased release of AVP can activate the discharge activity of AVP neurons, which is conducive to the release of AVP into the blood. In contrast, the synthesis rate of apelin in AVP neurons during dehydration exceeded its release rate, and plasma apelin level decreased, while apelin was accumulated in neurons. With reducing the release of apelin and attenuating the inhibitory effects of apelin on neurons, the discharge activity of AVP neurons was consequently enhanced (De Mota et al., 2004; Reaux-Le Goazigo et al., 2004). In addition to rodents, the same study that recruited 10 healthy volunteers (Azizi et al., 2008) revealed that IV injection of hypertonic saline caused the increase of plasma crystal osmotic pressure, while plasma AVP concentration increased and apelin concentration decreased. Conversely, water load decreased plasma AVP concentration and rapidly increased apelin level (Azizi et al., 2008). Thus, it can be concluded that plasma osmotic pressure is an important factor in determining plasma apelin level. In addition, apelin is negatively correlated with the secretion of AVP, and the antagonistic effects of apelin and AVP can protect the kidney against excessive water excretion, which is biologically significant in maintaining fluid balance.

An abnormal humoral status was found in APJ knockout mice. The amount of drinking water was significantly decreased in APJ knockout mice that could drink freely in spite of the fact that the urine volume and urine osmotic fluid remained normal. After 24 h of water shortage, the urine volume of wild-type mice was markedly reduced, and the urine osmotic pressure was significantly increased, whereas these changes did not occur in knockout mice. Although plasma AVP concentration was noticeably elevated in both wild-type and knockout mice after water deprivation, knockout mice were unable to respond to AVP. Further study revealed that the level of c-fos mRNA in the subfornical apparatus was decreased, the expression of PVN was increased, and the expression of the SON remained unchanged in APJ knockout mice when water was deprived (Roberts et al., 2009). The above-mentioned studies indicated that apelin/APJ system plays a pivotal role in water intake and discharge, as well as maintaining body fluid homeostasis.

### Regulatory Effects of Apelin/APJ System on Water Metabolism in Kidney Level

Apelin and its receptor APJ are expressed in all regions of kidneys of human and rat, suggesting that the regulatory effects of apelin on fluid homeostasis may be correlated to the kidney level. In the glomerulus, APJ mRNA is mainly expressed in the endothelial cells and VSMCs of the afferent and efferent arterioles (Huang et al., 2018). A previous study found that IV injection of apelin-17 could antagonize the vasoconstrictive effect caused by angiotensin II, and the specific mechanism may be associated with activating APJ of vascular endothelial cells to release NO, inhibiting the angiotensin II-mediated elevation of intracellular calcium concentration, causing vasodilation. After administration of NOS inhibitor L-NAME, the apelin-induced inhibition of calcium ions disappeared. Angiotensin II-induced vasoconstriction can be rapidly reversed by apelin, confirming the opposite vascular effect of apelin/angiotensin system (Galanth et al., 2012). In addition, apelin directly acts on APJ on VSMCs to mediate vasodilation, especially in renal medullary microcirculation (Hus-Citharel et al., 2008). Therefore, apelin participates in the regulation of renal hemodynamics by increasing renal blood flow and promoting urinary excretion to maintain body fluid homeostasis.

The APJ mRNA level is increased progressively along cortex to medullary collecting duct in rats, suggesting that apelin not only regulates water metabolism through CNS, but also has a direct effect on renal tubule and collecting ducts. This is the key site for AVP to exert antidiuretic effect through V2R. V2R is distributed in the basolateral membrane of renal collecting duct epithelial cells, and it activates PKA through the V2R-Gs protein-AC-cAMP pathway. PKA catalyzes the phosphorylation of aquaporin-2 (AQP-2) and embeds it into the apical membrane of collecting ducts to increase water permeability and produce antidiuretic effect (Gerbier et al., 2017). IV injection of apelin-17 into lactating rats could increase urine volume and significantly decrease urine osmotic pressure, while the secretion of sodium and potassium ions did not change remarkably. Immunohistochemistry analysis showed that AQP-2 expression in the apical membrane of rat outer medullary collecting duct epithelial cells was significantly reduced. It was reported that apelin-17 binding to APJ-coupled with Gi could lead to the decrease of cAMP production, in addition to the reduction of the number of AQP-2 located at the apical membrane of collecting duct cells, thereby reducing water reabsorption and exerting diuretic effect (Hus-Citharel et al., 2014). Additionally, apelin-17 could reduce the influx of extracellular calcium, which is crucial for the insertion of AQP-2 into the apical membrane. Studies have shown that cells loaded with the calcium chelator BAPTA could inhibit the transport of AQP-2 in the collecting duct cells. However, a number of scholars pointed out that cAMP alone is sufficient for intermembrane shuttling of AQP-2 (Lorenz et al., 2003; Boone and Deen, 2008; Hus-Citharel et al., 2014). Therefore, the role of calcium in the diuretic effect of apelin deserves further study.

In recent years, the effects of dimerization between APJ and other GPCRs on physiological functions have markedly attracted scholars’ attention. Given the co-localization and functional correlation of APJ and V2R in rat central and renal tubular epithelial cells, the existence of heterodimer between APJ and V2R was confirmed *in vivo* and *in vitro* by FRET, bioluminescence resonance energy transfer (BRET), and immunoprecipitation techniques. Whether the structure of the dimer is the main reason for the opposite biological effects of the two peptides, and the effects of the receptor dimer on ligand binding and intracellular signaling pathways require further investigations.

To date, due to the short half-life and relatively weak activity of apelin *in vivo*, derivatives or substitutes related to apelin have emerged (e.g., P92 and LIT01-196; Flahault et al., 2017; Gerbier et al., 2017). These derivatives could also inhibit the release of AVP, increase urine volume, and cause a significant and sustained decrease in blood pressure *in vivo*. They are more effective and possess longer half-life than natural apelin fragments. Therefore, these compounds are expected to be significant candidates for treatment of heart failure and hyponatremia.

## Elabela (A Novel Endogenous Peptidc Ligand of APJ)

Recently, a new endogenous peptidic ligand of APJ, namely Elabela, has been identified and shown to play a crucial role in embryonic development (Chng et al., 2013; Pauli et al., 2014). Apelin and elabela are endogenous ligands of angiotensin domain type 1 receptor-associated proteins (Chng et al., 2013). In addition, increasing evidences showed that elabela is also intimately associated with a large number of physiological processes in adulthood.

Elabela, a secreted peptide hormone, plays a pivotal role in heart morphogenesis, migration of mesendodermal cells, and endoderm differentiation (Chng et al., 2013). Elabela is expressed in undifferentiated human embryonic stem cells and is immediately downregulated during differentiation (Miura et al., 2004). Decreased cell growth, cell death, and loss of pluripotency in human embryonic stem cells (hESC) caused by the inhibition of elabela suggest that elabela acts as an important endogenous growth factor in human embryos (Ho et al., 2015) and has biologically functional effects on the growth of embryos (Chng et al., 2013; Pauli et al., 2014).

A role for elabela beyond development is described in adult human heart and blood vessels, where elabela is localized in endothelium (Chng et al., 2013). Elabela activates the APJ signaling pathways, promotes angiogenesis *via* disturbing the intracellular Ca^2+^ homeostasis (Cui et al., 2017) or acting through transform growth factor β (TGF-β/SMADs) pathway (Ho et al., 2015), and enhances vasodilation in mouse aorta (Wang et al., 2015) *via* suppressing ERK activation (Schreiber et al., 2017). More importantly, elabela inhibits angiotensin II-induced pressor effect in mouse (Yang et al., 2015).

Elabela also plays a key role in regulating water homeostasis. Elabela is detected in prostate and kidney (Wang et al., 2015). It regulates fluid homeostasis by binding to the APJ receptor to activate Gi signaling (Xu et al., 2018). Application of elabela, in line with apelin-17, has also shown a significant suppressive effect on urine osmolality and electrolyte excretion, e.g., concentrations of Na^+^ and K^+^ (Murza et al., 2016; Schreiber et al., 2017), while another study revealed that ICV injection of elabela could activate AVP (Santoso et al., 2015). Elabela may suppress food intake *via* activation of AVP and corticotropin-releasing hormone (CRH)-containing neurons, in addition to the increase of c-Fos expression and [Ca^2+^]i in PVN (Santoso et al., 2015). Furthermore, elabela may inhibit renal remodeling and possesses a direct anti-fibrotic effect. Elabela suppresses renal remodeling, decreases renal fibrosis, and reduces the expressions of fibrosis-associated genes in the kidneys of salt-induced hypertensive sprague-dawley (SD) rats *via* regulating TGF-β signaling pathway (Schreiber et al., 2017). A recent study demonstrated that elabela is essential for the skeletal development, bone formation, and bone homeostasis (Perez-Camps et al., 2016).

Collectively, increasing evidence has underscored the role of elabela in embryonic development, and in adulthood whereas little is known about functions of elabela in different pathophysiological events, as well as its precise molecular mechanism. Therefore, a comprehensive and systematic biological study on elabela needs to be carried out.

## Conclusion and Perspectives

**Figure 4:**
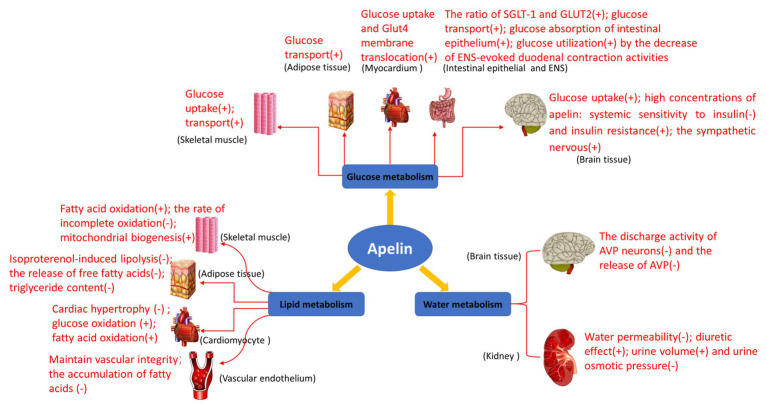
Biological functions of apelin in glucose metabolism, lipid metabolism, and water metabolism. Glucose metabolism: apelin promotes glucose uptake in skeletal muscle cells and adipose tissues, and increases overall glucose utilization; it also increases glucose uptake and Glut4 membrane translocation in mouse myocardium *in vivo*; it increases the net glucose flow of gastrointestinal mucosal barrier and enhances the glucose transport from intestinal lumen to blood in the intestinal epithelium; it improves glucose utilization by the decrease of enteric nervous system (ENS)-evoked duodenal contraction activities; and it regulates glucose homeostasis and promotes glucose uptake and utilization in the brain of the normally fed mice. High concentrations of apelin reduce systemic sensitivity to insulin and produce insulin resistance, activate the sympathetic nervous system through reactive oxygen species (ROS) signaling pathways, and promote hepatic glycogenolysis and gluconeogenesis in the brain of obese or diabetic mice. Lipid metabolism: apelin increases perilipin content around lipid vesicles and reduces free fatty acid (FFA) release from adipocytes by inhibiting lipolysis. Apelin also increases fatty acid oxidation in skeletal muscle and myocardium, reducing the rate of incomplete oxidation of long-chain acylcarnitines associated with insulin. In addition, apelin may prevent the development of obesity and atherosclerosis by reducing the release of free fatty acids and increasing perilipin content around lipid vesicles, and prevents fatty deposits in the blood vessels and plaque formation. Water metabolism: the expression of apelin/APJ in magnocellular neurons of the hypothalamus and periventricular organelles suggests that it may be involved in the regulation of body fluid balance. Apelin and its receptor APJ are expressed in all regions of human and rat kidneys, regulating body fluid homeostasis at the kidney level.

The biological study of apelin/APJ system in human and animals is becoming more and more clear. Its main functions include enhancing myocardial contractility, stimulating angiogenesis, relaxing blood vessels to reduce blood pressure, enhancing glucose uptake and utilization by tissue cells, inhibiting lipolysis, and enhancing diuresis (Figure 4). Additionally, maybe apelin/APJ system exerts various beneficial or unfavorable pathophysiological effects during the occurrence and development of a variety of diseases including obesity, diabetes mellitus, syndrome of inappropriate antidiuretic hormone secretion, and other diseases (Boucher et al., 2005; Heinonen et al., 2005; Li et al., 2006; Dray et al., 2008; Valle et al., 2008; Gerbier et al., 2017; Boulkeroua et al., 2019). Apelin/APJ system may be expected to become a therapeutic target for cardiovascular diseases (e.g., hypertension and heart failure). Compared with the traditional diuretics, apelin exerts a positive inotropic effect on the heart, while increases water excretion. Therefore, the drugs targeting apelin/APJ system undoubtedly may provide more treatment options for patients with congestive heart failure accompanied with hyponatremia. To provide more precise guidance for the development of clinical drugs, further detailed studies are warranted on the metabolism and signaling pathways associated with apelin/APJ system.

## Author Contributions

GH: writing most of the contents of this review. ZW and RZ: adding some content to the review. XC and WS: checking and revising the paper. All authors contributed to the article and approved the submitted version.

### Conflict of Interest

The authors declare that the research was conducted in the absence of any commercial or financial relationships that could be construed as a potential conflict of interest.

## References

[ref1] Alfarano C. Foussal C. Lairez O. Calise D. Attané C. Anesia R.. (2015). Transition from metabolic adaptation to maladaptation of the heart in obesity: role of apelin. Int. J. Obes. 39, 312–320. 10.1038/ijo.2014.122, PMID: 25027224 PMC4326962

[ref2] Attané C. Daviaud D. Dray C. Dusaulcy R. Masseboeuf M. Prévot D.. (2011). Apelin stimulates glucose uptake but not lipolysis in human adipose tissue ex vivo. J. Mol. Endocrinol. 46, 21–28. 10.1677/JME-10-0105, PMID: 21062936

[ref3] Azizi Y. Faghihi M. Imani A. Roghani M. Nazari A. (2013). Post-infarct treatment with [Pyr1]-apelin-13 reduces myocardial damage through reduction of oxidative injury and nitric oxide enhancement in the rat model of myocardial infarction. Peptides 46, 76–82. 10.1016/j.peptides.2013.05.006, PMID: 23727032

[ref4] Azizi M. Iturrioz X. Blanchard A. Peyrard S. De Mota N. Chartrel N.. (2008). Reciprocal regulation of plasma apelin and vasopressin by osmotic stimuli. J. Am. Soc. Nephrol. 19, 1015–1024. 10.1681/ASN.2007070816, PMID: 18272843 PMC2386722

[ref5] Bełtowski J. (2006). Apelin and visfatin: unique “beneficial” adipokines upregulated in obesity? Med. Sci. Monit. 12, Ra112–Ra119. PMID: 16733497

[ref6] Bertrand C. Valet P. Castan-Laurell I. (2015). Apelin and energy metabolism. Front. Physiol. 6:115. 10.3389/fphys.2015.00115, PMID: 25914650 PMC4392293

[ref7] Bodineau L. Taveau C. Hh L. Q. S. Osterstock G. Queguiner I. Moos F.. (2011). Data supporting a new physiological role for brain apelin in the regulation of hypothalamic oxytocin neurons in lactating rats. Endocrinology 152, 3492–3503. 10.1210/en.2011-0206, PMID: 21733827

[ref8] Boone M. Deen P. M. (2008). Physiology and pathophysiology of the vasopressin-regulated renal water reabsorption. Pflugers Arch. 456, 1005–1024. 10.1007/s00424-008-0498-1, PMID: 18431594 PMC2518081

[ref9] Boucher J. Masri B. Daviaud D. Gesta S. Guigné C. Mazzucotelli A.. (2005). Apelin, a newly identified adipokine up-regulated by insulin and obesity. Endocrinology 146, 1764–1771. 10.1210/en.2004-1427, PMID: 15677759

[ref10] Boulkeroua C. Ayari H. Khalfaoui T. Lafrance M. Besserer-Offroy É. Ekindi N.. (2019). Apelin-13 regulates vasopressin-induced aquaporin-2 expression and trafficking in kidney collecting duct cells. Cell. Physiol. Biochem. 53, 687–700. 10.33594/000000165, PMID: 31577078

[ref11] Brailoiu G. C. Dun S. L. Yang J. Ohsawa M. Chang J. K. Dun N. J. (2002). Apelin-immunoreactivity in the rat hypothalamus and pituitary. Neurosci. Lett. 327, 193–197. 10.1016/S0304-3940(02)00411-1, PMID: 12113910

[ref12] Breen D. M. Rasmussen B. A. Côté C. D. Jackson V. M. Lam T. K. (2013). Nutrient-sensing mechanisms in the gut as therapeutic targets for diabetes. Diabetes 62, 3005–3013. 10.2337/db13-0523, PMID: 23970519 PMC3749331

[ref13] Castan-Laurell I. Dray C. Attané C. Duparc T. Knauf C. Valet P. (2011). Apelin, diabetes, and obesity. Endocrine 40, 1–9. 10.1007/s12020-011-9507-9, PMID: 21725702

[ref14] Chandrasekharan B. Srinivasan S. (2007). Diabetes and the enteric nervous system. Neurogastroenterol. Motil. 19, 951–960. 10.1111/j.1365-2982.2007.01023.x, PMID: 17971027 PMC3711013

[ref15] Chaves-Almagro C. Castan-Laurell I. Dray C. Knauf C. Valet P. Masri B. (2015). Apelin receptors: from signaling to antidiabetic strategy. Eur. J. Pharmacol. 763, 149–159. 10.1016/j.ejphar.2015.05.017, PMID: 26007641

[ref16] Chen X. Bai B. Tian Y. Du H. Chen J. (2014). Identification of serine 348 on the apelin receptor as a novel regulatory phosphorylation site in apelin-13-induced G protein-independent biased signaling. J. Biol. Chem. 289, 31173–31187. 10.1074/jbc.M114.574020, PMID: 25271156 PMC4223320

[ref17] Chng S. C. Ho L. Tian J. Reversade B. (2013). ELABELA: a hormone essential for heart development signals via the apelin receptor. Dev. Cell 27, 672–680. 10.1016/j.devcel.2013.11.002, PMID: 24316148

[ref18] Chun H. J. Ali Z. A. Kojima Y. Kundu R. K. Sheikh A. Y. Agrawal R.. (2008). Apelin signaling antagonizes Ang II effects in mouse models of atherosclerosis. J. Clin. Invest. 118, 3343–3354. 10.1172/JCI34871, PMID: 18769630 PMC2525695

[ref19] Clarke K. J. Whitaker K. W. Reyes T. M. (2009). Diminished metabolic responses to centrally-administered apelin-13 in diet-induced obese rats fed a high-fat diet. J. Neuroendocrinol. 21, 83–89. 10.1111/j.1365-2826.2008.01815.x, PMID: 19076266

[ref20] Cui C. Merritt R. Fu L. Pan Z. (2017). Targeting calcium signaling in cancer therapy. Acta Pharm. Sin. B 7, 3–17. 10.1016/j.apsb.2016.11.001, PMID: 28119804 PMC5237760

[ref21] D’Aniello C. Lonardo E. Iaconis S. Guardiola O. Liguoro A. M. Liguori G. L.. (2009). G protein-coupled receptor APJ and its ligand apelin act downstream of Cripto to specify embryonic stem cells toward the cardiac lineage through extracellular signal-regulated kinase/p70S6 kinase signaling pathway. Circ. Res. 105, 231–238. 10.1161/CIRCRESAHA.109.201186, PMID: 19574549

[ref22] De Mota N. Lenkei Z. Llorens-Cortès C. (2000). Cloning, pharmacological characterization and brain distribution of the rat apelin receptor. Neuroendocrinology 72, 400–407. 10.1159/000054609, PMID: 11146423

[ref23] De Mota N. Reaux-Le Goazigo A. El Messari S. Chartrel N. Roesch D. Dujardin C.. (2004). Apelin, a potent diuretic neuropeptide counteracting vasopressin actions through inhibition of vasopressin neuron activity and vasopressin release. Proc. Natl. Acad. Sci. U. S. A. 101, 10464–10469. 10.1073/pnas.0403518101, PMID: 15231996 PMC478592

[ref24] Delaere F. Magnan C. Mithieux G. (2010). Hypothalamic integration of portal glucose signals and control of food intake and insulin sensitivity. Diabetes Metab. 36, 257–262. 10.1016/j.diabet.2010.05.001, PMID: 20561808

[ref25] Devic E. Paquereau L. Vernier P. Knibiehler B. Audigier Y. (1996). Expression of a new G protein-coupled receptor X-msr is associated with an endothelial lineage in *Xenopus laevis*. Mech. Dev. 59, 129–140. 10.1016/0925-4773(96)00585-0, PMID: 8951791

[ref26] Diano S. Liu Z. W. Jeong J. K. Dietrich M. O. Ruan H. B. Kim E.. (2011). Peroxisome proliferation-associated control of reactive oxygen species sets melanocortin tone and feeding in diet-induced obesity. Nat. Med. 17, 1121–1127. 10.1038/nm.2421, PMID: 21873987 PMC3388795

[ref27] Dray C. Knauf C. Daviaud D. Waget A. Boucher J. Buléon M.. (2008). Apelin stimulates glucose utilization in normal and obese insulin-resistant mice. Cell Metab. 8, 437–445. 10.1016/j.cmet.2008.10.003, PMID: 19046574

[ref28] Dray C. Sakar Y. Vinel C. Daviaud D. Masri B. Garrigues L.. (2013). The intestinal glucose-apelin cycle controls carbohydrate absorption in mice. Gastroenterology 144, 771–780. 10.1053/j.gastro.2013.01.004, PMID: 23313268

[ref29] Drougard A. Duparc T. Brenachot X. Carneiro L. Gouazé A. Fournel A.. (2014). Hypothalamic apelin/reactive oxygen species signaling controls hepatic glucose metabolism in the onset of diabetes. Antioxid. Redox Signal. 20, 557–573. 10.1089/ars.2013.5182, PMID: 23879244 PMC3901354

[ref30] Drougard A. Fournel A. Marlin A. Meunier E. Abot A. Bautzova T.. (2016). Central chronic apelin infusion decreases energy expenditure and thermogenesis in mice. Sci. Rep. 6:31849. 10.1038/srep31849, PMID: 27549402 PMC4994119

[ref31] Duparc T. Colom A. Cani P. D. Massaly N. Rastrelli S. Drougard A.. (2011a). Central apelin controls glucose homeostasis via a nitric oxide-dependent pathway in mice. Antioxid. Redox Signal. 15, 1477–1496. 10.1089/ars.2010.3454, PMID: 21395477

[ref32] Duparc T. Naslain D. Colom A. Muccioli G. G. Massaly N. Delzenne N. M.. (2011b). Jejunum inflammation in obese and diabetic mice impairs enteric glucose detection and modifies nitric oxide release in the hypothalamus. Antioxid. Redox Signal. 14, 415–423. 10.1089/ars.2010.3330, PMID: 20879900

[ref33] Edinger A. L. Hoffman T. L. Sharron M. Lee B. Yi Y. Choe W.. (1998). An orphan seven-transmembrane domain receptor expressed widely in the brain functions as a coreceptor for human immunodeficiency virus type 1 and simian immunodeficiency virus. J. Virol. 72, 7934–7940. 10.1128/JVI.72.10.7934-7940.1998, PMID: 9733831 PMC110125

[ref34] El Messari S. Iturrioz X. Fassot C. De Mota N. Roesch D. Llorens-Cortes C. (2004). Functional dissociation of apelin receptor signaling and endocytosis: implications for the effects of apelin on arterial blood pressure. J. Neurochem. 90, 1290–1301. 10.1111/j.1471-4159.2004.02591.x, PMID: 15341513

[ref35] Fan W. Boston B. A. Kesterson R. A. Hruby V. J. Cone R. D. (1997). Role of melanocortinergic neurons in feeding and the agouti obesity syndrome. Nature 385, 165–168. 10.1038/385165a0, PMID: 8990120

[ref36] Flahault A. Couvineau P. Alvear-Perez R. Iturrioz X. Llorens-Cortes C. (2017). Role of the vasopressin/apelin balance and potential use of metabolically stable apelin analogs in water metabolism disorders. Front. Endocrinol. 8:120. 10.3389/fendo.2017.00120, PMID: 28620355 PMC5450005

[ref37] Fournel A. Drougard A. Duparc T. Marlin A. Brierley S. M. Castro J.. (2017). Apelin targets gut contraction to control glucose metabolism via the brain. Gut 66, 258–269. 10.1136/gutjnl-2015-310230, PMID: 26565000 PMC5284480

[ref38] Fukaya M. Mizuno A. Arai H. Muto K. Uebanso T. Matsuo K.. (2007). Mechanism of rapid-phase insulin response to elevation of portal glucose concentration. Am. J. Physiol. Endocrinol. Metab. 293, E515–E522. 10.1152/ajpendo.00536.2006, PMID: 17473051

[ref39] Galanth C. Hus-Citharel A. Li B. Llorens-Cortès C. (2012). Apelin in the control of body fluid homeostasis and cardiovascular functions. Curr. Pharm. Des. 18, 789–798. 10.2174/138161212799277770, PMID: 22236125

[ref40] Gerbier R. Alvear-Perez R. Margathe J. F. Flahault A. Couvineau P. Gao J.. (2017). Development of original metabolically stable apelin-17 analogs with diuretic and cardiovascular effects. FASEB J. 31, 687–700. 10.1096/fj.201600784R, PMID: 27815337

[ref41] Gerbier R. Leroux V. Couvineau P. Alvear-Perez R. Maigret B. Llorens-Cortes C.. (2015). New structural insights into the apelin receptor: identification of key residues for apelin binding. FASEB J. 29, 314–322. 10.1096/fj.14-256339, PMID: 25359495

[ref42] Grill H. J. Ginsberg A. B. Seeley R. J. Kaplan J. M. (1998). Brainstem application of melanocortin receptor ligands produces long-lasting effects on feeding and body weight. J. Neurosci. 18, 10128–10135. 10.1523/JNEUROSCI.18-23-10128.1998, PMID: 9822766 PMC6793290

[ref43] Habata Y. Fujii R. Hosoya M. Fukusumi S. Kawamata Y. Hinuma S.. (1999). Apelin, the natural ligand of the orphan receptor APJ, is abundantly secreted in the colostrum. Biochim. Biophys. Acta 1452, 25–35. 10.1016/s0167-4889(99)00114-7, PMID: 10525157

[ref44] He S. Li J. Wang J. Zhang Y. (2019). Hypoxia exposure alleviates impaired muscular metabolism, glucose tolerance, and aerobic capacity in apelin-knockout mice. FEBS Open Bio 9, 498–509. 10.1002/2211-5463.12587, PMID: 30868058 PMC6396165

[ref45] Heinonen M. V. Purhonen A. K. Miettinen P. Pääkkönen M. Pirinen E. Alhava E.. (2005). Apelin, orexin-a and leptin plasma levels in morbid obesity and effect of gastric banding. Regul. Pept. 130, 7–13. 10.1016/j.regpep.2005.05.003, PMID: 15970339

[ref46] Higuchi K. Masaki T. Gotoh K. Chiba S. Katsuragi I. Tanaka K.. (2007). Apelin, an APJ receptor ligand, regulates body adiposity and favors the messenger ribonucleic acid expression of uncoupling proteins in mice. Endocrinology 148, 2690–2697. 10.1210/en.2006-1270, PMID: 17347313

[ref47] Ho L. Tan S. Y. Wee S. Wu Y. Tan S. J. Ramakrishna N. B.. (2015). ELABELA is an endogenous growth factor that sustains hESC self-renewal via the PI3K/AKT pathway. Cell Stem Cell 17, 435–447. 10.1016/j.stem.2015.08.010, PMID: 26387754

[ref48] Hosoya M. Kawamata Y. Fukusumi S. Fujii R. Habata Y. Hinuma S.. (2000). Molecular and functional characteristics of APJ. Tissue distribution of mRNA and interaction with the endogenous ligand apelin. J. Biol. Chem. 275, 21061–21067. 10.1074/jbc.M908417199, PMID: 10777510

[ref49] Huang Z. Wu L. Chen L. (2018). Apelin/APJ system: a novel potential therapy target for kidney disease. J. Cell. Physiol. 233, 3892–3900. 10.1002/jcp.26144, PMID: 28796300

[ref50] Hus-Citharel A. Bodineau L. Frugière A. Joubert F. Bouby N. Llorens-Cortes C. (2014). Apelin counteracts vasopressin-induced water reabsorption via cross talk between apelin and vasopressin receptor signaling pathways in the rat collecting duct. Endocrinology 155, 4483–4493. 10.1210/en.2014-1257, PMID: 25157454

[ref51] Hus-Citharel A. Bouby N. Frugière A. Bodineau L. Gasc J. M. Llorens-Cortes C. (2008). Effect of apelin on glomerular hemodynamic function in the rat kidney. Kidney Int. 74, 486–494. 10.1038/ki.2008.199, PMID: 18509323

[ref52] Hwangbo C. Wu J. Papangeli I. Adachi T. Sharma B. Park S.. (2017). Endothelial APLNR regulates tissue fatty acid uptake and is essential for apelin’s glucose-lowering effects. Sci. Transl. Med. 9:eaad4000. 10.1126/scitranslmed.aad4000, PMID: 28904225 PMC5703224

[ref53] Kang Y. Kim J. Anderson J. P. Wu J. Gleim S. R. Kundu R. K.. (2013). Apelin-APJ signaling is a critical regulator of endothelial MEF2 activation in cardiovascular development. Circ. Res. 113, 22–31. 10.1161/CIRCRESAHA.113.301324, PMID: 23603510 PMC3739451

[ref54] Kawamata Y. Habata Y. Fukusumi S. Hosoya M. Fujii R. Hinuma S.. (2001). Molecular properties of apelin: tissue distribution and receptor binding. Biochim. Biophys. Acta 1538, 162–171. 10.1016/s0167-4889(00)00143-9, PMID: 11336787

[ref55] Kleinz M. J. Baxter G. F. (2008). Apelin reduces myocardial reperfusion injury independently of PI3K/Akt and P70S6 kinase. Regul. Pept. 146, 271–277. 10.1016/j.regpep.2007.10.002, PMID: 18022257

[ref56] Kleinz M. J. Davenport A. P. (2005). Emerging roles of apelin in biology and medicine. Pharmacol. Ther. 107, 198–211. 10.1016/j.pharmthera.2005.04.001, PMID: 15907343

[ref57] Knauf C. Cani P. D. Ait-Belgnaoui A. Benani A. Dray C. Cabou C.. (2008). Brain glucagon-like peptide 1 signaling controls the onset of high-fat diet-induced insulin resistance and reduces energy expenditure. Endocrinology 149, 4768–4777. 10.1210/en.2008-0180, PMID: 18556349

[ref58] Knauf C. Drougard A. Fournel A. Duparc T. Valet P. (2013). Hypothalamic actions of apelin on energy metabolism: new insight on glucose homeostasis and metabolic disorders. Horm. Metab. Res. 45, 928–934. 10.1055/s-0033-1351321, PMID: 23950038

[ref59] Lam C. K. Chari M. Lam T. K. (2009). CNS regulation of glucose homeostasis. Physiology 24, 159–170. 10.1152/physiol.00003.2009, PMID: 19509126

[ref60] Lee D. K. Cheng R. Nguyen T. Fan T. Kariyawasam A. P. Liu Y.. (2000). Characterization of apelin, the ligand for the APJ receptor. J. Neurochem. 74, 34–41. 10.1046/j.1471-4159.2000.0740034.x, PMID: 10617103

[ref61] Lee D. K. Ferguson S. S. George S. R. O’dowd B. F. (2010). The fate of the internalized apelin receptor is determined by different isoforms of apelin mediating differential interaction with beta-arrestin. Biochem. Biophys. Res. Commun. 395, 185–189. 10.1016/j.bbrc.2010.03.151, PMID: 20353754

[ref62] Lee D. K. Jeong J. H. Oh S. Jo Y. H. (2015). Apelin-13 enhances arcuate POMC neuron activity via inhibiting M-current. PLoS One 10:e0146210. 10.1371/journal.pone.0146210, PMID: 25782002 PMC4363569

[ref63] Li Y. Chen J. Bai B. Du H. Liu Y. Liu H. (2012). Heterodimerization of human apelin and kappa opioid receptors: roles in signal transduction. Cell. Signal. 24, 991–1001. 10.1016/j.cellsig.2011.12.012, PMID: 22200678

[ref64] Li L. Yang G. Li Q. Tang Y. Yang M. Yang H.. (2006). Changes and relations of circulating visfatin, apelin, and resistin levels in normal, impaired glucose tolerance, and type 2 diabetic subjects. Exp. Clin. Endocrinol. Diabetes 114, 544–548. 10.1055/s-2006-948309, PMID: 17177135

[ref65] Lin F. Wu H. Chen H. Xin Z. Yuan D. Wang T.. (2014). Molecular and physiological evidences for the role in appetite regulation of apelin and its receptor APJ in Ya-fish (*Schizothorax prenanti*). Mol. Cell. Endocrinol. 396, 46–57. 10.1016/j.mce.2014.08.009, PMID: 25150624

[ref66] Lorenz D. Krylov A. Hahm D. Hagen V. Rosenthal W. Pohl P.. (2003). Cyclic AMP is sufficient for triggering the exocytic recruitment of aquaporin-2 in renal epithelial cells. EMBO Rep. 4, 88–93. 10.1038/sj.embor.embor711, PMID: 12524527 PMC1315811

[ref67] Maguire J. J. Kleinz M. J. Pitkin S. L. Davenport A. P. (2009). [Pyr1]apelin-13 identified as the predominant apelin isoform in the human heart: vasoactive mechanisms and inotropic action in disease. Hypertension 54, 598–604. 10.1161/HYPERTENSIONAHA.109.134619, PMID: 19597036

[ref68] Masri B. Morin N. Pedebernade L. Knibiehler B. Audigier Y. (2006). The apelin receptor is coupled to Gi1 or Gi2 protein and is differentially desensitized by apelin fragments. J. Biol. Chem. 281, 18317–18326. 10.1074/jbc.M600606200, PMID: 16679320

[ref69] Medhurst A. D. Jennings C. A. Robbins M. J. Davis R. P. Ellis C. Winborn K. Y.. (2003). Pharmacological and immunohistochemical characterization of the APJ receptor and its endogenous ligand apelin. J. Neurochem. 84, 1162–1172. 10.1046/j.1471-4159.2003.01587.x, PMID: 12603839

[ref70] Miura T. Luo Y. Khrebtukova I. Brandenberger R. Zhou D. Thies R. S.. (2004). Monitoring early differentiation events in human embryonic stem cells by massively parallel signature sequencing and expressed sequence tag scan. Stem Cells Dev. 13, 694–715. 10.1089/scd.2004.13.694, PMID: 15684837

[ref71] Murza A. Sainsily X. Coquerel D. Côté J. Marx P. Besserer-Offroy É.. (2016). Discovery and structure-activity relationship of a bioactive fragment of ELABELA that modulates vascular and cardiac functions. J. Med. Chem. 59, 2962–2972. 10.1021/acs.jmedchem.5b01549, PMID: 26986036

[ref72] O’Carroll A. M. Lolait S. J. Harris L. E. Pope G. R. (2013). The apelin receptor APJ: journey from an orphan to a multifaceted regulator of homeostasis. J. Endocrinol. 219, R13–R35. 10.1530/JOE-13-0227, PMID: 23943882

[ref73] O’Carroll A. M. Selby T. L. Palkovits M. Lolait S. J. (2000). Distribution of mRNA encoding B78/APJ, the rat homologue of the human APJ receptor, and its endogenous ligand apelin in brain and peripheral tissues. Biochim. Biophys. Acta 1492, 72–80. 10.1016/s0167-4781(00)00072-5, PMID: 11004481

[ref74] O’Dowd B. F. Heiber M. Chan A. Heng H. H. Tsui L. C. Kennedy J. L.. (1993). A human gene that shows identity with the gene encoding the angiotensin receptor is located on chromosome 11. Gene 136, 355–360. 10.1016/0378-1119(93)90495-O, PMID: 8294032

[ref75] Ostrowski N. L. Lolait S. J. Bradley D. J. O’carroll A. M. Brownstein M. J. Young W. S. 3rd. (1992). Distribution of V1a and V2 vasopressin receptor messenger ribonucleic acids in rat liver, kidney, pituitary and brain. Endocrinology 131, 533–535. 10.1210/endo.131.1.1535312, PMID: 1535312

[ref76] Pauli A. Norris M. L. Valen E. Chew G. L. Gagnon J. A. Zimmerman S.. (2014). Toddler: an embryonic signal that promotes cell movement via Apelin receptors. Science 343:1248636. 10.1126/science.1248636, PMID: 24407481 PMC4107353

[ref77] Perez-Camps M. Tian J. Chng S. C. Sem K. P. Sudhaharan T. Teh C.. (2016). Quantitative imaging reveals real-time Pou5f3-Nanog complexes driving dorsoventral mesendoderm patterning in zebrafish. eLife 5:e11475. 10.7554/eLife.11475, PMID: 27684073 PMC5042653

[ref78] Puigserver P. (2005). Tissue-specific regulation of metabolic pathways through the transcriptional coactivator PGC1-alpha. Int. J. Obes. 29 (Suppl. 1), S5–S9. 10.1038/sj.ijo.0802905, PMID: 15711583

[ref79] Qiu J. Fang Y. Rønnekleiv O. K. Kelly M. J. (2010). Leptin excites proopiomelanocortin neurons via activation of TRPC channels. J. Neurosci. 30, 1560–1565. 10.1523/JNEUROSCI.4816-09.2010, PMID: 20107083 PMC3095824

[ref80] Qiu J. Zhang C. Borgquist A. Nestor C. C. Smith A. W. Bosch M. A.. (2014). Insulin excites anorexigenic proopiomelanocortin neurons via activation of canonical transient receptor potential channels. Cell Metab. 19, 682–693. 10.1016/j.cmet.2014.03.004, PMID: 24703699 PMC4183666

[ref81] Reaux A. De Mota N. Skultetyova I. Lenkei Z. El Messari S. Gallatz K.. (2001). Physiological role of a novel neuropeptide, apelin, and its receptor in the rat brain. J. Neurochem. 77, 1085–1096. 10.1046/j.1471-4159.2001.00320.x, PMID: 11359874

[ref82] Reaux-Le Goazigo A. Bodineau L. De Mota N. Jeandel L. Chartrel N. Knauf C.. (2011). Apelin and the proopiomelanocortin system: a new regulatory pathway of hypothalamic α-MSH release. Am. J. Physiol. Endocrinol. Metab. 301, E955–E966. 10.1152/ajpendo.00090.2011, PMID: 21846903

[ref83] Reaux-Le Goazigo A. Morinville A. Burlet A. Llorens-Cortes C. Beaudet A. (2004). Dehydration-induced cross-regulation of apelin and vasopressin immunoreactivity levels in magnocellular hypothalamic neurons. Endocrinology 145, 4392–4400. 10.1210/en.2004-0384, PMID: 15166125

[ref84] Roberts E. M. Newson M. J. Pope G. R. Landgraf R. Lolait S. J. O’carroll A. M. (2009). Abnormal fluid homeostasis in apelin receptor knockout mice. J. Endocrinol. 202, 453–462. 10.1677/JOE-09-0134, PMID: 19578099 PMC2729781

[ref85] Roberts E. M. Pope G. R. Newson M. J. Landgraf R. Lolait S. J. O’carroll A. M. (2010). Stimulus-specific neuroendocrine responses to osmotic challenges in apelin receptor knockout mice. J. Neuroendocrinol. 22, 301–308. 10.1111/j.1365-2826.2010.01968.x, PMID: 20136689

[ref86] Santoso P. Maejima Y. Kumamoto K. Takenoshita S. Shimomura K. (2015). Central action of ELABELA reduces food intake and activates arginine vasopressin and corticotropin-releasing hormone neurons in the hypothalamic paraventricular nucleus. Neuroreport 26, 820–826. 10.1097/WNR.0000000000000431, PMID: 26237243

[ref87] Schreiber C. A. Holditch S. J. Generous A. Ikeda Y. (2017). Sustained ELABELA gene therapy in high-salt diet-induced hypertensive rats. Curr. Gene Ther. 16, 349–360. 10.2174/1566523217666161121111906, PMID: 27903222

[ref88] Seale P. Kajimura S. Yang W. Chin S. Rohas L. M. Uldry M.. (2007). Transcriptional control of brown fat determination by PRDM16. Cell Metab. 6, 38–54. 10.1016/j.cmet.2007.06.001, PMID: 17618855 PMC2564846

[ref89] Sunter D. Hewson A. K. Dickson S. L. (2003). Intracerebroventricular injection of apelin-13 reduces food intake in the rat. Neurosci. Lett. 353, 1–4. 10.1016/S0304-3940(03)00351-3, PMID: 14642423

[ref90] Szokodi I. Tavi P. Földes G. Voutilainen-Myllylä S. Ilves M. Tokola H.. (2002). Apelin, the novel endogenous ligand of the orphan receptor APJ, regulates cardiac contractility. Circ. Res. 91, 434–440. 10.1161/01.res.0000033522.37861.69, PMID: 12215493

[ref91] Tatemoto K. Hosoya M. Habata Y. Fujii R. Kakegawa T. Zou M. X.. (1998). Isolation and characterization of a novel endogenous peptide ligand for the human APJ receptor. Biochem. Biophys. Res. Commun. 251, 471–476. 10.1006/bbrc.1998.9489, PMID: 9792798

[ref92] Than A. Cheng Y. Foh L. C. Leow M. K. Lim S. C. Chuah Y. J.. (2012). Apelin inhibits adipogenesis and lipolysis through distinct molecular pathways. Mol. Cell. Endocrinol. 362, 227–241. 10.1016/j.mce.2012.07.002, PMID: 22842084

[ref93] Than A. He H. L. Chua S. H. Xu D. Sun L. Leow M. K.. (2015). Apelin enhances brown adipogenesis and browning of white adipocytes. J. Biol. Chem. 290, 14679–14691. 10.1074/jbc.M115.643817, PMID: 25931124 PMC4505534

[ref94] Valle A. Hoggard N. Adams A. C. Roca P. Speakman J. R. (2008). Chronic central administration of apelin-13 over 10 days increases food intake, body weight, locomotor activity and body temperature in C57BL/6 mice. J. Neuroendocrinol. 20, 79–84. 10.1111/j.1365-2826.2007.01617.x, PMID: 18081555

[ref95] Wang Z. Yu D. Wang M. Wang Q. Kouznetsova J. Yang R.. (2015). Elabela-apelin receptor signaling pathway is functional in mammalian systems. Sci. Rep. 5:8170. 10.1038/srep08170, PMID: 25639753 PMC4313117

[ref96] Xu J. Chen L. Jiang Z. Li L. (2018). Biological functions of Elabela, a novel endogenous ligand of APJ receptor. J. Cell. Physiol. 233, 6472–6482. 10.1002/jcp.26492, PMID: 29350399 PMC13482497

[ref97] Xu S. Han P. Huang M. Wu J. C. Chang C. Tsao P. S.. (2012). In vivo, ex vivo, and in vitro studies on apelin’s effect on myocardial glucose uptake. Peptides 37, 320–326. 10.1016/j.peptides.2012.08.004, PMID: 22906703

[ref98] Yamada T. Katagiri H. (2007). Avenues of communication between the brain and tissues/organs involved in energy homeostasis. Endocr. J. 54, 497–505. 10.1507/endocrj.KR-106, PMID: 17510499

[ref99] Yamamoto T. Habata Y. Matsumoto Y. Yasuhara Y. Hashimoto T. Hamajyo H.. (2011). Apelin-transgenic mice exhibit a resistance against diet-induced obesity by increasing vascular mass and mitochondrial biogenesis in skeletal muscle. Biochim. Biophys. Acta 1810, 853–862. 10.1016/j.bbagen.2011.05.004, PMID: 21609753

[ref100] Yang P. Maguire J. J. Davenport A. P. (2015). Apelin, Elabela/toddler, and biased agonists as novel therapeutic agents in the cardiovascular system. Trends Pharmacol. Sci. 36, 560–567. 10.1016/j.tips.2015.06.002, PMID: 26143239 PMC4577653

[ref101] Yue P. Jin H. Aillaud M. Deng A. C. Azuma J. Asagami T.. (2010). Apelin is necessary for the maintenance of insulin sensitivity. Am. J. Physiol. Endocrinol. Metab. 298, E59–E67. 10.1152/ajpendo.00385.2009, PMID: 19861585 PMC2806109

[ref102] Yue P. Jin H. Xu S. Aillaud M. Deng A. C. Azuma J.. (2011). Apelin decreases lipolysis via G(q), G(i), and AMPK-dependent mechanisms. Endocrinology 152, 59–68. 10.1210/en.2010-0576, PMID: 21047945 PMC3033059

[ref103] Zhou N. Fan X. Mukhtar M. Fang J. Patel C. A. Dubois G. C.. (2003a). Cell-cell fusion and internalization of the CNS-based, HIV-1 co-receptor, APJ. Virology 307, 22–36. 10.1016/s0042-6822(02)00021-1, PMID: 12667811

[ref104] Zhou N. Zhang X. Fan X. Argyris E. Fang J. Acheampong E.. (2003b). The N-terminal domain of APJ, a CNS-based coreceptor for HIV-1, is essential for its receptor function and coreceptor activity. Virology 317, 84–94. 10.1016/j.virol.2003.08.026, PMID: 14675627

[ref105] Zhu S. Sun F. Li W. Cao Y. Wang C. Wang Y.. (2011). Apelin stimulates glucose uptake through the PI3K/Akt pathway and improves insulin resistance in 3T3-L1 adipocytes. Mol. Cell. Biochem. 353, 305–313. 10.1007/s11010-011-0799-0, PMID: 21461612

